# Broad cross protection by recombinant live attenuated influenza H3N2 seasonal virus expressing conserved M2 extracellular domain in a chimeric hemagglutinin

**DOI:** 10.1038/s41598-021-83704-0

**Published:** 2021-02-18

**Authors:** Bo Ryoung Park, Ki-Hye Kim, Tatiana Kotomina, Min-Chul Kim, Young-Man Kwon, Subbiah Jeeva, Yu-Jin Jung, Noopur Bhatnagar, Irina Isakova-Sivak, Daria Mezhenskaya, Larisa Rudenko, Bao-Zhong Wang, Sang-Moo Kang

**Affiliations:** 1 Institute for Biomedical Sciences, Georgia State University, Atlanta, GA 30303 USA; 2 Department of Virology, Institute of Experimental Medicine, St Petersburg, Russia; 3 CARESIDE Co., Ltd., Seongnam, Gyeonggi-do Republic of Korea

**Keywords:** Immunology, Microbiology, Diseases, Molecular medicine

## Abstract

Hemagglutinin (HA)-based current vaccines provide suboptimum cross protection. Influenza A virus contains an ion channel protein M2 conserved extracellular domain (M2e), a target for developing universal vaccines. Here we generated reassortant influenza virus rgH3N2 4xM2e virus (HA and NA from A/Switzerland/9715293/2013/(H3N2)) expressing chimeric 4xM2e-HA fusion proteins with 4xM2e epitopes inserted into the H3 HA N-terminus. Recombinant rgH3N2 4xM2e virus was found to retain equivalent growth kinetics as rgH3N2 in egg substrates. Intranasal single inoculation of mice with live rgH3N2 4xM2e virus was effective in priming the induction of M2e specific IgG antibody responses in mucosal and systemic sites as well as T cell responses. The rgH3N2 4xM2e primed mice were protected against a broad range of different influenza A virus subtypes including H1N1, H3N2, H5N1, H7N9, and H9N2. The findings support a new approach to improve the efficacy of current vaccine platforms by recombinant influenza virus inducing immunity to HA and cross protective M2e antigens.

## Introduction

Influenza virus causes one of the most common respiratory diseases in humans, resulting in significant public health concerns and deaths annually worldwide^1,2^. Influenza A virus is a negative-sense single-stranded RNA virus containing 8 segmented genomes, belonging to the *Orthomyxoviridae* family, and has an antigenic variety from 18 subtypes (H1–H18) of hemagglutinin (HA) and 11 subtypes (N1–N11) of neuraminidase (NA)^3^. Antigenic diversity is a challenging difficulty in preventing influenza. Vaccination has been the most effective preventive measure against influenza virus infection. The most common licensed platforms are inactivated influenza and live-attenuated influenza vaccines (LAIV).

The overall vaccine effectiveness during 2005–2018 seasons is a wide range of low efficacy between 10 and 60% as estimated in the US Flu Vaccine Effectiveness Network^4^. Due to emergence of drifting mutations in circulating H3N2 strains, the vaccine effectiveness against H3N2 was estimated to be 6% during the 2014–2015 season^5,6^. To overcome continued antigenic changes, universal vaccination strategies have been focused on inducing immunity to conserved epitopes and domains present in all influenza A viruses, including the M2 extracellular domain epitopes (M2e)^7,8^ and the HA stalk domains^9,10^.

Different platforms and vaccine adjuvants have been investigated to overcome low immunogenicity of M2e epitopes. M2e-based vaccine candidates include Hepatitis B virus core protein conjugates (M2e-HBc) with adjuvants^11,12^, virus-like particles (VLP) presenting M2e tandem repeats (5xM2e VLP)^13^, and flagellin conjugates (4.M2e-tFliC)^14^ and fusion with oligomer stabilizing domains (M2e-tGCN4)^15^. M2e expressing viral vectored vaccines were reported using adenovirus^16^, modified vaccinia virus Ankara^17^, and a T7-bacteriophage^18^. These previous strategies inducing M2e immunity alone were insufficient for conferring optimum protection and incompatible with current vaccine platforms. No universal vaccine against influenza is on the market.

Vaccination of combined M2e VLP and inactivated influenza vaccines induced both cross protective M2e and strain specific HA immunity^19,20^. To enhance the cross protective efficacy by a strategy of utilizing current vaccine platforms, recombinant influenza H1N1 virus A/Puerto Rico/8/1934 (A/PR8) was engineered to express chimeric 4xM2e-HA where tandem M2e epitopes were inserted in the N-terminus HA^21^. A chimeric HA with a single M2e in the head site Ca was tested in inactivated recombinant A/PR8 virus inducing cross protection^22^. However, the protection was not tested against a wide range of different subtypes.

In recent years, antigenic drifts have severely limited the effectiveness of the H3N2 component of seasonal influenza vaccines. Here, using the reverse genetic (rg) technique, we generated reassortant seasonal influenza rgH3N2 4xM2e virus containing chimeric 4xM2e-HA in which the HA and NA genes were derived from A/Switzerland/9715293/2013 (H3N2) and the remaining 6 genes from the A/PR8 backbone. Reassortant rgH3N2 4xM2e virus containing chimeric 4xM2e-HA was found to retain comparable growth properties but display highly attenuated phenotypes in mice. Intranasal single inoculation of mice with rgH3N2 4xM2e virus could effectively induce a broad range of enhanced cross protection against different influenza A virus subtypes including H1N1, H3N2, H5N1, H7N9, and H9N2. This study implicates a strategy of improving cross protection by utilizing currently licensed recombinant influenza vaccine platforms.

## Results

### Generation of reassortant influenza H3N2 virus vaccine containing 4xM2e in an HA conjugate

**Figure 1:**
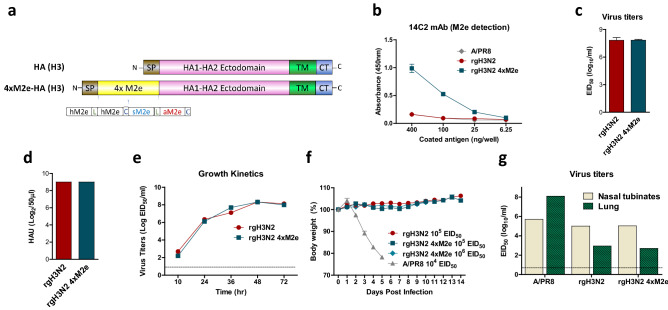
Reassortants rgH3N2 and rgH3N2 4xM2e virus expressing chimeric 4xM2e-HA are attenuated in vivo*.* (**a**) Diagram for chimeric 4xM2e-HA (H3) structures. The sequence H3 HA was derived from A/Switzerland/2013 (H3N2). SP: signal peptide, tandem repeat 4xM2e is composed of human, swine, and avian influenza A viruses. hM2e: SLLTEVETPIRNEWGSRSNDSSD, sM2e: SLLTEVETPTRSEWESRSSDSSD, aM2e: SLLTEVETPTRNEWESRSSDSSD. L and C represent linker (AAAGGAA) and connector (AAAPGAA) respectively. (**b**) 14C2 M2e specific mAb ELISA assays were performed to characterize 6:2 reassortants rgH3N2 and rgH3N2 4xM2e viruses (4 µg/ ELISA plate well) generated by reverse genetics (rg) using the A/PR8 backbone. (**c**) Infectious virus titer averages (EID_50_) of rgH3N2 (2 replicates) and rgH3N2 4xM2e virus (3 replicates) preparations in egg substrates. (**d**) Hemagglutination activity units (HAU, log_2_/50 µl) for rgH3N2 and rgH3N2 4xM2e viruses using chicken red blood cells. There was no variation in these two replicates that showed the same results on HAU assays. (**e**) Growth kinetics of reassortant viruses in embryonated chicken eggs at 33 °C infected with 10^4^ EID_50_ of rgH3N2 and rgH3N2 4xM2e. (**c**–**e**) A representative out of 2 repeats. (**f**) Body weight changes in BALB/c mice (n = 10, too small variations to be shown) IN inoculated with reassortant rgH3N2 viruses (10^5^–10^6^ EID_50_) or A/PR8 (10^4^ EID_50_). (**g**) Infectious virus titers (EID_50_) in nasal turbinates and lung extracts from mice (n = 3, pooled) at 3-day post infection with A/PR8 (10^4^ EID_50_), rgH3N2 (10^6^ EID_50_) and rgH3N2 4xM2e (10^6^ EID_50_).

Current strain specific HA-based influenza vaccine is less effective in conferring cross protection. M2e has been targeted to induce broad but weak cross protection. To induce immunity against both HA and highly conserved M2e epitopes, a seasonal A/Switzerland/2013 H3 HA gene conjugated with four tandem M2e (4xM2e) repeat was constructed (Fig. 1a and Supplementary Table S1). Rescued viruses expressing wild type (WT) HA (rgH3N2) or chimeric HA (rgH3N2 4xM2e) were amplified in eggs and harvested. Antigenic characterization by ELISA showed that rgH3N2 4xM2e virus was highly reactive to M2e specific mAb 14C2 whereas rgH3N2 and A/PR8 virus controls did not show such M2e reactivity (Fig. 1b). Both rgH3N2 and rgH3N2 4xM2e viruses displayed high antigenic reactivity to mouse antisera of rgH3N2 infection and goat antisera of A/Indiana/2011 (H3N2) immunization (Supplementary Fig. S1). These results support that 6:2 reassortant rgH3N2 4xM2e virus displays high reactivity to M2e specific 14C2 mAb and retains similar antigenicity to antisera of H3N2 viruses as compared to rgH3N2 virus.

### Reassortant rgH3N2 4xM2e virus is attenuated in mice but does not compromise replication capacity in egg substrates

We determined whether rgH3N2 4xM2e virus would retain the comparable replication capacity in egg substrates, compared to WT rgH3N2. Both chimeric rgH3N2 4xM2e and WT rgH3N2 viruses displayed similarly high levels of egg infectious titers (EID_50_/ml) and hemagglutination activity units (HAU) (Fig. 1c,d). Next, at designated time points after incubation of embryonated chicken eggs with 10^4^ EID_50_ of rgH3N2 or rgH3N2 4xM2e, in vitro growth kinetics of infectious titers (EID_50_) were determined (Fig. 1e). The results showed that rgH3N2 4xM2e virus exhibited an approximately equivalent growth kinetics in egg substrates as WT rgH3N2 virus. The reassortant rgH3N2 4xM2e virus does not compromise replication capacity in egg substrates. Consistent, the overall trends in the growth patterns of rgH3N2 and rgH3N2 4xM2 reassortants in MDCK cells were similarly observed as those in the eggs despite some variations at different culture times and temperatures (Supplementary Fig. S2).

As an indicator of pathogenicity, weight changes and activity were monitored in mice after intranasal inoculation with rgH3N2 4xM2e and rgH3N2 viruses. Both mouse groups infected with rgH3N2 (10^5^ EID_50_) and rgH3N2 4xM2e (10^5^ and 10^6^ EID_50_) did not show body weight loss (Fig. 1f) while the mice with A/PR8 even at a 100-fold lower dose (10^4^ EID_50_) consistently displayed severe weight loss and died of infection. Consistent, mice inoculated with WT A/PR8 virus showed highest levels of replicating viral titers in the lower respiratory tracts of lungs, which is at approximately 100 folds higher than those in the upper respiratory nasal turbinates at day 3 post inoculation (10^4^ EID_50_) (Fig. 1g). In contrast, rgH3N2 (10^6^ EID_50_) and rgH3N2 4xM2e (10^6^ EID_50_) viruses showed approximately 10^5^ magnitudes lower lung viral titers than WT A/PR8 virus (Fig. 1g). Instead, 100 folds higher viral replications were observed in the nasal turbinates than those in the lungs after inoculation of mice with rgH3N2 and rgH3N2 4xM2e virus (Fig. 1g). Consistent with the differences in viral titers between in the upper (nasal) and lower (lung) respiratory tracts, virus growths of reassortants at 33 °C MDCK cell cultures were 1 to 2 log10 magnitudes higher than those at 37 °C MDCK cell cultures (Supplementary Fig. S2). These results suggest that reassortant rgH3N2 and chimeric rgH3N2 4xM2e viruses are highly attenuated in mice with restricted replication in the upper respiratory tracts but retains replication capacity in egg or MDCK cell substrates.

### Prime dose of live rgH3N2 4xM2e virus induces M2e specific IgG isotype antibody responses

**Figure 2:**
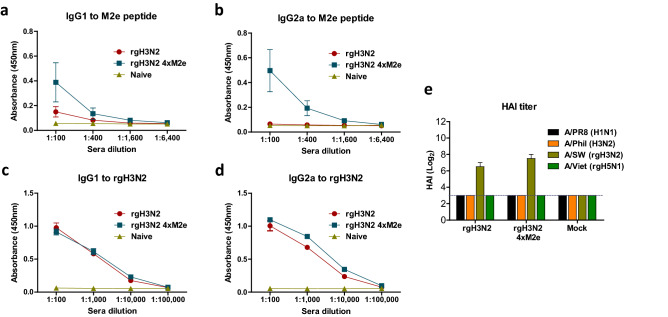
Intranasal prime inoculation of live rgH3N2 4xM2e virus induces M2e specific serum IgG isotype antibody responses. (**a**) M2e specific IgG1 antibodies. (**b**) M2e specific IgG2a antibodies. (**c**, **d**) Virus rgH3N2 specific IgG1 and IgG2a antibodies. Serum samples were collected at 2 weeks after prime dose intranasal inoculation of mice (n = 15 /group). (**e**) Serum HAI titers against the viruses (A/PR8, A/Phil, rgH3N2 and A/Viet) were determined 14 days after immunization. rgH3N2: 6:2 reassortant with WT HA and NA of A/Switzerland/2013. rgH3N2 4xM2e: 6:2 reassortant with 4XM2e-HA and NA of A/Switzerland/2013. Each individual mouse animal is analyzed. Error bars indicate mean ± SEM.

Intranasal prime inoculation of mice with live rgH3N2 4xM2e virus (10^5^ EID_50_) induced M2e specific IgG1 and IgG2a antibody responses at significantly higher levels than rgH3N2 (Fig. 2a,b). IgG antibodies specific for human M2e were induced at higher levels in prime sera from rgH3N2 4xM2e vaccination than those specific for avian M2e or swine M2e (Supplementary Fig. S3a), suggesting possible contribution of the extra 2 × hM2e in the rgH3N2 4xM2e construct. Both rgH3N2 and rgH3N2 4xM2e immunized groups showed similarly high levels of IgG isotype antibodies specific for H3N2 virus (Fig. 2c,d). Both sera from rgH3N2 and rgH3N2 4xM2e immune mice exhibited vaccine strain specific HAI activities against rgH3N2 (A/Switzerland) virus (Fig. 2e) but not against A/PR8 (H1N1), A/Philippines (A/Phil H3N2) and A/Vietnam (A/Viet rgH5N1). Therefore, prime dose of live rgH3N2 4xM2e but not rgH3N2 virus could induce M2e specific IgG isotype antibodies in mice.

### Prime dose of live rgH3N2 4xM2e vaccination provides broad cross protection

**Figure 3:**
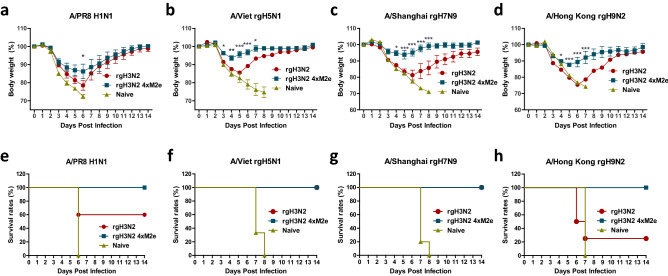
A single dose of live rgH3N2 4xM2e virus provides enhanced heterosubtypic cross protection against different subtype influenza A viruses. Weight changes and survival rates in primed mice after challenge with (**a**, **e**) H1N1 A/PR8 (3 LD_50_), (**b**, **f**) rgH5N1 A/Vietnam (5 LD_50_), (**c**, **g**) rgH7N9 virus (reassortant A/Shanghai, 6 LD_50_) and (**d**, **h**) rgH9N2 virus (reassortant A/Hong Kong, 20 LD_50_). Differential challenge doses were used depending on the pathogenicity and HA phylogenic distance of virus. Groups of mice (n = 5 or 6) were intranasally primed with rgH3N2 (10^5^ EID_50_) or rgH3N2 4xM2e (10^5^ EID_50_) and then challenged 3 weeks later. Two independent repeat of mouse challenge experiments confirms the reproducibility of data. rgH3N2: 6:2 reassortant with WT HA and NA of A/Switzerland/2013. rgH3N2 4xM2e: 6:2 reassortant with 4XM2e-HA and NA of A/Switzerland/2013. Each individual mouse animal is analyzed. Error bars show mean ± SEM. The statistical significances between rgH3N2 group versus rgH3N2 4xM2e group were determined using two-way ANOVA and indicated in *, *P* < 0.05; **, *P* < 0.01; ***, *P* < 0.001.

Intranasally prime vaccinated mice with 10^5^ EID_50_ of rgH3N2 or rgH3N2 4xM2e were challenged with different subtype influenza viruses including H1N1 (A/PR8), rgPR8 with avian M2 (rgPR8 Mm, rgPR8 Mg), H3N2 (A/Phil), rgH5N1 (A/Viet), rgH7N9 (A/Shanghai), and rgH9N2 (A/Hong Kong). The rgH3N2 4xM2e group showed higher survival rates (100%) and a trend of less weight loss against A/PR8 challenge, compared to the rgH3N2 group with 60% survival rates (Fig. 3a,e). Next, we tested PR8 reassortants containing avian M2 (rgPR8 Mm, rgPR8 Mg, Supplementary Fig. S4a, b, d, e) as a challenge virus, following single-dose vaccination with rgH3N2 or rgH3N2 4xM2e. A trend of less weight loss against rgPR8 Mm (M gene from A/Mandarin duck/Korea/PSC24-24/2010, H5N1) or rgPR8 Mg (M gene from A/Chicken/Korea/Gimje/2008, H5N1) was observed in the rgH3N2 4xM2e group, compared to those in the rgH3N2 control group. When challenged with a wild type 2009 H1N1 pandemic A/California/04/09 virus containing swine M2, the rgH3N2 4xM2e primed mice showed a pattern of less weight loss and quicker recovery than the rgH3N2 group (Supplementary Fig. S5). However, these difference between these two groups were not statistically significant.

To test the breadth of cross protection, different subtypes of PR8 reassortants were used as a challenge virus. The rgH3N2 4xM2e primed mice exhibited significantly less weight loss (~ 5%) than rgH3N2 primed mice (~ 16%) at day 5 after challenge with rgH5N1 (Fig. 3b,f). Similarly, when challenged with rgH7N9 virus, the rgH3N2 4xM2e group displayed average weight loss 6% compared to 19% weight loss in rgH3N2 prime mice and the mock control with over 20% weight loss and 0% survival rates (Fig. 3c,g). After a high lethal dose (100 LD_50_) challenge with rgH9N2 virus, rgH3N2 4xM2e primed mice showed 75% survival protection whereas rgH3N2 immune and mock control mice all died of infection (Supplementary Fig. S4c, f). When challenged with rgH9N2 at 20 LD_50_ dose, rgH3N2 4xM2e primed mice showed better protection as evidenced by significantly preventing weight loss (average 11% day 6) and enhancing survival rates than rgH3N2 immune mice displaying 25% weight loss (day 6) and 25% survival rates (Fig. 3d,h).

**Figure 4:**
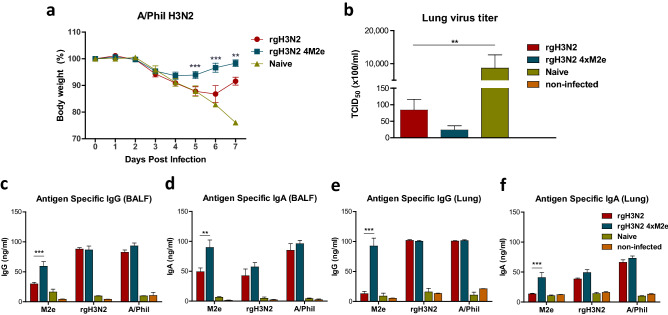
Prime dose rgH3N2 4xM2e provides enhanced protection against heterologous H3N2 A/Phil virus and M2e specific IgG and IgA antibodies in mucosal sites. The mice primed with rgH3N2 or rgH3N2 4xM2e were challenged with heterologous A/Phil H3N2 virus (50 LD_50_). A relatively high challenge dose was used because of the same subtype virus. (**a**) Body weight changes after A/Phil challenge. Two independent repeat of mouse challenge experiments confirms the reproducibility of data. (**b**) Lung viral titers (TCID_50_ × 100/ml) at 5-day post challenge with A/Phil. (**c**–**f**) IgG and IgA antibody responses specific for M2e or viral (rgH3N2, A/Phil) antigens in BALF and lung extracts at 5 days after challenge. ELISA viral antigens are rgH3N2 and A/Phil. Error bars indicate mean value ± SEM. The statistical significances between rgH3N2 group versus rgH3N2 4xM2e group were determined using two-way ANOVA (**a**, **c**–**e**) or one-way ANOVA (**b**) and indicated in *, *P* < 0.05; **, *P* < 0.01; ***, *P* < 0.001.

After challenge with A/Phil (H3N2), 3% weight loss was observed with rgH3N2 4xM2e primed mice in contrast to 12% weight loss was observed with rgH3N2 primed mice (Fig. 4a). Collectively, these results suggest that single dose intranasal priming of mice with rgH3N2 4xM2e could provide enhanced cross protection against diverse subtypes of influenza A viruses containing human or avian M2.

### Priming with rgH3N2 4xM2e virus results in enhanced responses of M2e specific IgG and IgA antibodies in mucosal respiratory and systemic sites upon challenge

Consistently, the mice that received a single dose (10^5^ EID_50_) of rgH3N2 4xM2e showed more effective cross protection against A/Phil H3N2 virus as evidenced by less weight loss compared to the mice with rgH3N2 (Fig. 4a). At day 5 post infection with A/Phil, naive mice showed the highest levels of lung virus titers (~ 10^6^ TCID_50_) as determined in MDCK cells (Fig. 4b). Approximately 100 folds lower lung viral titers (8 × 10^3^ TCID_50_) were observed in the rgH3N2 group than those in naïve mice, which are approximately 3.5-fold higher titers than those (2.3 × 10^3^ TCID_50_) in the rgH3N2 4xM2e group (Fig. 4b). There was no statistical significance in lung viral titers between the rgH3N2 and rgH3N2 4xM2e group (Fig. 4b) and a similar pattern of viral titers was detected when determined by EID_50_ in egg substrates.

Significantly higher levels of M2e specific IgG (Fig. 4c,e) and IgA (Fig. 4d,f) antibodies were induced in BALF (Fig. 4c,d) and lung extracts (Fig. 4e,f) from the rgH3N2 4xM2e group compared to the rgH3N2 group at 5 days after infection with A/Phil. Meanwhile, virus (rgH3N2, A/Phil) specific IgG and IgA antibodies were similarly induced in BALF and lung extracts at high levels in the both rgH3N2 4xM2e and rgH3N2 groups (Fig. 4c–f).

**Figure 5:**
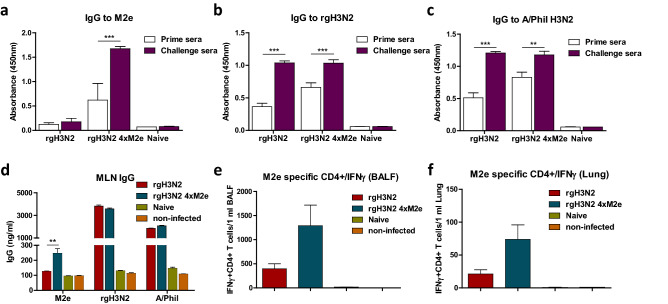
Single inoculation of rgH3N2 4xM2e virus effectively primes M2e and virus specific immune responses. Sera after rgH3N2 or rgH3N2 4xM2e immunization (day 14) and challenge with A/Phil H3N2 virus (50 LD_50_, day7) were collected respectively to determine IgG antibody response specific for (**a**) M2e (1:100 sera), (**b**) rgH3N2 (1:10,000 sera), and (**c**) A/Phil (H3N2) (1:10,000 sera) antigens. (**d**) In vitro production of M2e, rgH3N2, and A/Phil specific IgG antibodies in mediastinal lymph node (MLN) cell cultures. (**e**–**f**) Flow cytometry of intracellular cytokine staining for detection of IFN-γ secreting CD4^+^ T cells specific for M2e from BALF (**e**) and lung cells (**f)** at day 5 (**d**–**f**) or 7 (**a**–**c**) following A/Phil challenge. IFN-γ^+^ CD4^+^ T cells were presented from total BALF (1 ml) and Lung (1 ml) cells from individual mouse. Error bars indicate mean ± SEM. The statistical significances between rgH3N2 group versus rgH3N2 4xM2e group were determined using two-way ANOVA and indicated in *, *P* < 0.05; **, *P* < 0.01; ***, *P* < 0.001.

We determined recall immune responses by measuring M2e and virus specific IgG at day 5 or 7 post challenge with heterologous A/Phil virus (Fig. 5). The rgH3N2 4xM2e primed mice showed significantly increased M2e specific IgG levels in sera (threefold in OD values) and draining lymph nodes MLN after challenge (Fig. 5a,d) while the other groups did not. The increased IgG levels specific for vaccine rgH3N2 (Fig. 5b) and challenge virus A/Phil (Fig. 5c) were similarly observed in post-challenge sera from both groups. Intracellular cytokine staining results indicate the induction of M2e-stimulated CD4 T cells secreting IFN-γ at high frequencies (Fig. 5e,f) and relatively low numbers of IFN-γ^+^ CD8 T cells (Supplementary Fig. S6) in BALF and lung cells from the rgH3N2 4xM2e primed mice. These results suggest that the levels of M2e specific antibodies and T cell immunity induced by priming rgH3N2 4xM2e in mucosal (BALF, lungs) and systemic (sera, MLN) sites might have contributed to cross-protection.

### Depletion of CD4 and CD8 T cells results in differential cross protection between rgH3N2 4xM2e and rgH3N2 prime vaccination

**Figure 6:**
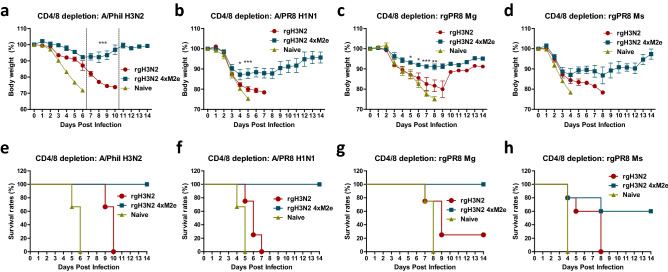
Depletion of CD4 and CD8 T cells results in differential cross protection between rgH3N2 and rgH3N2 4xM2e primed mice. The rgH3N2 or rgH3N2 4xM2e primed mice (n = 4 or 5/group) were treated with α-CD4/α-CD8 antibodies for T cell depletion prior to influenza virus infection. (**a**–**d**) Weight changes and (**e**–**h**) survival rates were monitored followed by A/Phil (H3N2) (17 LD_50_), A/PR8 (1.5 LD_50_), rgPR8 Mg (3 LD_50_) and rgPR8 Ms (10 LD_50_) challenge, respectively. Differential challenge doses were used depending on the subtype, pathogenicity, and T cell-depletion. Error bars indicate mean ± SEM. The statistical significances between rgH3N2 group versus rgH3N2 4xM2e group were determined using two-way ANOVA and indicated in *, *P* < 0.05; ***, *P* < 0.001.

We observed substantial cross protection by priming rgH3N2 virus even without the induction of M2e specific antibodies and cross HAI activities, suggesting the roles of cross protective T cells. Also, to determine whether M2e specific antibodies would significantly contribute to cross protection, rgH3N2 or rgH3N2 4xM2e primed mice were treated with T cell depleting anti-CD4 or anti-CD8 antibodies prior to challenge with 25 LD_50_ of A/Phil (H3N2). As shown in supplementary data (Supplementary Fig. S7), T cells in the groups immunized with rgH3N2 or rgH3N2 4xM2e were effectively depleted by delivering either anti-CD4 or anti-CD8 or both antibodies prior to infection with A/Phil (H3N2, 17 LD_50_). Anti-CD4 (Supplementary Fig. S8a,c) or anti-CD8 (Supplementary Fig. S8b,d) antibody treatment resulted in significant weight loss (24% or 16% respectively) in the rgH3N2 group, lowering the survival rate (50% with anti-CD4, Supplementary Fig. S8c), compared to moderate weight loss (9.5 to 13%) in the rgH3N2 4xM2e group. Statistically significant difference was observed in the CD4 depleted but not CD8 depleted rgH3N2 4xM2e group prior to challenge, suggesting that CD4 is primarily responsible. When both CD4 and CD8 were depleted, the rgH3N2 group displayed significant weight loss and all mice reaching the humane endpoint (Fig. 6a,e). In contrast, 100% mice from the rgH3N2 4xM2e group survived with 8% body weight loss (Fig. 6a,e). These results suggest that M2e antibodies contribute to cross protection by a prime dose of rgH3N2 4xM2e regardless of T cells at the time of challenge whereas CD4 and CD8 T cells particularly CD4 T cells might play a significant role in cross protection by rgH3N2.

We extended the impact of both CD4 and CD8 depletion on cross protection against A/PR8 (H1N1) and PR8 reassortants with avian M2 (Fig. 6). Treatment of rgH3N2 4xM2e primed mice with CD4 and CD8 depleting antibodies resulted in 100% survival rates and approximately 13% weight loss after A/PR8 (H1N1) challenge whereas rgH3N2 primed mice showed over 20% weight loss and 0% survival rates (Fig. 6b,f). With CD4 and CD8 T cell depletion, the significant differences in weight changes and survival rates between the groups were observed after rgPR8 Mg challenge (Fig. 6c,g). While rgH3N2 4xM2e mice lost 9% of body weight with 100% survival rate, rgH3N2 mice had 20% weight loss with 20% survival rates. In consistent, the rgH3N2 4xM2e group showed significantly enhanced survival protection with less weight loss against a high lethal dose (20 LD_50_) of rgPR8 Ms virus (M gene from A/Shanghai/2/2013, H7N9) compared to the rgH3N2 group with 0% survival (Fig. 6d,h). The weight recovery was delayed in surviving mice with CD4 and CD8 T cell depletion after challenge, suggesting an important role of T cells in recovery or preventing severe viral pathology. These results support the differential roles of CD4 and CD8 T cells and M2e specific IgG antibodies in conferring cross protection by rgH3N2 and rgH3N2 4xM2e prime immunization.

### Boost dose of rgH3N2 4xM2e further enhances M2e antibodies

**Figure 7:**
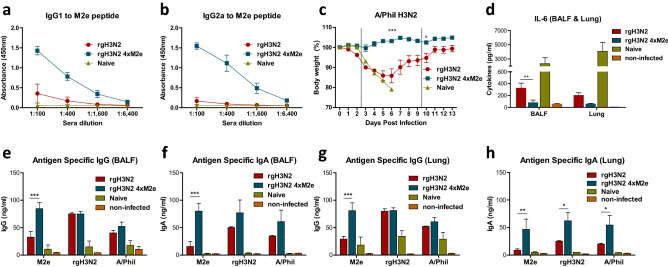
Boost dose of rgH3N2 4xM2e enhances cross protection against A/Phil (H3N2) challenge, correlating with M2e specific IgG levels and lower inflammatory cytokine. Sera were collected at 2 weeks after boost immunization of primed mice with rgH3N2 or rgH3N2 4xM2e. (**a**) IgG1 or (**b**) IgG2a antibody responses to M2e peptide. The rgH3N2 or rgH3N2 4xM2e boosted mice (n = 4 /group) were treated with α-CD4/α-CD8 (10 µg/10 µg per individual) antibodies for T cell depletion prior to A/Phil (H3N2) challenge. (**c**) Weight changes of rgH3N2 or rgH3N2 4xM2e prime-boost mice after challenge with A/Phil (200 LD_50_). (**d**) IL-6 levels in BALF or lung extracts at day 5 post challenge with A/Phil. ELISA of IgG or IgA antibodies specific for M2e peptide and inactivated virus antigens (rgH3N2 or A/Phil) in (**e**, **f**) BALF and (**g**, **h**) lung extracts at day 5 post challenge with A/Phil. Error bars indicate mean ± SEM. The statistical significances between rgH3N2 group versus rgH3N2 4xM2e group were determined using two-way ANOVA and indicated in *, *P* < 0.05; **, *P* < 0.01; ***, *P* < 0.001.

Boost inoculation (10^6^ EID_50_ rgH3N2 4xM2e) induced significantly higher levels of M2e specific IgG1 and IgG2a isotype antibodies (Fig. 7a,b) than post prime IgG antibody levels (Fig. 2a,b). Consistent with post prime, hM2e IgG antibody levels post boost with rgH3N2 4xM2e were significantly higher than those of swine M2e or avian M2e antibodies (Supplementary Fig. S3b). Both rgH3N2 and rgH3N2 4xM2e groups increased IgG isotype antibodies and HAI activity against vaccine strain rgH3N2 but not against heterologous viruses including A/PR8, A/Phil, and rgH5N1 (Supplementary Fig. S9a, b, c) compared to prime sera.

### Boost dose of rgH3N2 4xM2e enhances cross protection and correlates with higher M2e specific IgG levels and lower inflammatory cytokines in the respiratory sites

At 3 weeks after boost dose, rgH3N2 and rgH3N2 4xM2e immunized mice were treated with T cell depleting anti-CD4 and anti-CD8 antibodies and then challenged with A/Phil H3N2 at a high dose. The rgH3N2 4xM2e group showed 100% protection without weight loss whereas the rgH3N2 group displayed substantial weight loss (15%) after A/Phil challenge (Fig. 7c). Consistent with efficacy of cross protection, inflammatory cytokine IL-6 levels were low in BALF and lung extracts from the rgH3N2 4xM2e group (Fig. 7d). Significantly higher levels of M2e specific IgG and IgA antibodies in BALF and lung extracts were induced in the rgH3N2 4xM2e group than those in the rgH3N2 group after A/Phil challenge (Fig. 7e–h). Also, the levels of IgA antibodies specific for rgH3N2 and A/Phil viruses were higher in lung extracts from the rgH3N2 4xM2e group than those from the rgH3N2 group (Fig. 7h).

**Figure 8:**
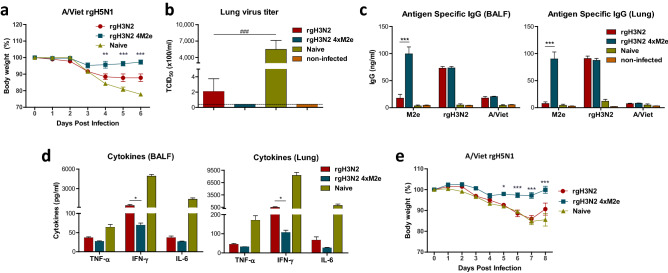
Boost with rgH3N2 4xM2e enhances cross protection against heterosubtypic A/Viet rgH5N1 virus by vaccination and immune sera. (**a**, **b**) Heterosubtypic cross protective efficacy of boost immune mice after challenge with rgH5N1 (A/Vietnam, 50 LD_50_). (**a**) Weight changes of boost immunized mice (n = 5 /group) after rgH5N1 challenge. (**b**) Lung viral titers (TCID_50_ × 100/ml) at 6-day post challenge with rgH5N1. (**c**) ELISA of IgG antibodies specific for M2e, rgH3N2, or rgH5N1 in BALF and lung extracts at day 6 post challenge with A/Viet rgH5N1. ELISA viral antigens are M2e peptide, rgH3N2 or A/Viet. (**d**) Inflammatory cytokines in BALF and lung extracts at day 6 post challenge with A/Viet rgH5N1. (**e**) Roles of boost immune sera in cross protection against rgH5N1 virus as determined by monitoring weight changes. Naïve mice (n = 3 /group) were intranasally inoculated with rgH5N1 (2 LD_50_) virus mixed with boost immune sera (rgH3N2, rgH3N2 4xM2e) or unvaccinated naïve sera. Error bars indicate mean ± SEM. The statistical significances between rgH3N2 group versus rgH3N2 4xM2e group were determined using two-way ANOVA (**a**, **c**–**e**) or one-way ANOVA (**b**) and indicated in *, *P* < 0.05; **, *P* < 0.01; ***, *P* < 0.001; ^###^, *P* < 0.0005.

Consistently, boost dose of rgH3N2 4xM2e also enhanced cross protection against heterosubtypic rgH5N1 virus, as shown by less weight loss (~ 4% versus 13% in rgH3N2 control) and undetectable lung viral loads (Fig. 8a,b). Particularly IgG levels specific for M2e were induced at significantly higher levels in BALF and lung extracts from the rgH3N2 4xM2e group than those in the rgH3N2 group or naïve group (Fig. 8c). IgG levels to vaccine (rgH3N2) and challenge virus (rgH5N1) were similar in both groups. As expected, the rgH3N2 and rgH3N2 4xM2e groups presented significantly lower amounts of TNF-α, IFN-γ and IL-6 than the naïve group after infection with rgH5N1 (Fig. 8d). Moreover, rgH3N2 4xM2e vaccine group exhibited lower levels of proinflammatory cytokines (IFN-γ) compared to the rgH3N2 group.

To determine the role and capability of immune sera in inducing cross protection against heterosubtypic rgH5N1 virus, naïve mice were intranasally infected with A/Vietnam virus (2 LD_50_) after mixing with boost immune sera from the rgH3N2 and rgH3N2 4xM2e group, and naïve sera respectively (Fig. 8e). The group with rgH3N2 4xM2e immune sera displayed significantly less weight loss (~ 3%) compared to the rgH3N2 serum group with substantial weight loss (~ 15%) similar to the naïve serum group (Fig. 8e). At day 8 post challenge with rgH5N1 virus, the rgH3N2 group started to recover (Fig. 8e). Taken together, these results indicate that M2e specific antibodies in mucosal and systemic sites provide cross protection by restricting viral replication and preventing inflammation.

## Discussion

The induction of strain specific neutralizing antibodies is the main immunity by current influenza vaccination, which is suboptimal for providing cross-protection. M2 is incorporated into influenza virions at a very low level^23^ and poorly immunogenic after vaccination or even with live virus infection^24,25^. Several strategies were reported in an attempt to overcome the poor immunogenicity of M2e, including fusion of M2e peptides to immunogenic carrier proteins and use of adjuvants^11,26,27^ or delivery of VLPs containing M2e epitopes^13,18,28^. M2e fusion protein vaccines (M2e-HBc, M2e-flagellins) were tested in Phase I/II clinical trials, resulting in high seroconversion inducing M2e specific antibody responses^29,30^. Intramuscular vaccination with M2e fusion protein vaccines was well tolerated except for the high doses (3 µg or 10 µg) of M2e-flagellin conjugates in healthy individuals^31^. However, there has been no further clinical advancement of M2e based vaccines probably due to low efficacy of M2e immunity alone^19,32,33^. One drawback might be short-lived M2e antibodies. Another disadvantage is the non-neutralizing nature of M2e immunity conferring low efficacy. An approach to overcome these drawbacks of M2e fusion protein vaccines would be to develop chimeric influenza virus vaccines where multi M2e epitopes are fused to the HA molecules in a way retaining HA functional and immunogenic integrity. It is expected that the tolerability and protective efficacy of chimeric influenza virus vaccines would be compatible with seasonal vaccines in addition to providing extra M2e immunity. In this approach, we generated recombinant seasonal H3N2 influenza A virus expressing chimeric H3 HA molecules with heterologous tandem repeat 4xM2e epitopes. This recombinant H3N2 virus with chimeric 4xM2e-HA was found to be highly attenuated, effective in inducing both M2e and HA immunity, conferring cross protection against different viruses H1N1, reassortants with avian M2, H3N2, H5N1, H7N9, and H9N2 subtypes in mice with a single dose.

The comparable in vitro growth kinetics of rgH3N2 4xM2e viruses suggest the integrity of HA functions in recombinant H3N2 virus containing chimeric H3 4xM2e-HA. The pathogenicity of rgH3N2 reassortants (up to 10^6^ EID_50_) with the PR8 backbone was highly attenuated in mice by 1,000 to 10,000 folds in the aspects of weight changes and viral replication (10^3^ versus 10^8^ EID_50_ titers) in the lungs compared to the WT A/PR8 (H1N1) virus (10^4^ EID_50_). The attenuated rgH3N2 phenotypes might serve as an appropriate platform to test vaccine candidates in mice. Owning to the attenuated phenotypes, a single prime dose of rgH3N2 4xM2e within a typical range (10^5^–10^6^ EID_50_) for LAIV vaccination^34,35^ could mimic vaccination without displaying pathogenic phenotypes. Chimeric rgH3N2 4xM2e was not defective in inducing immunity to virus.

It is significant to observe substantial levels of IgG isotype antibodies specific for M2e after prime with recombinant seasonal rgH3N2 4xM2e virus compared to WT rgH3N2 virus. The rgH3N2 4xM2e primed mice were protected against H1N1 A/PR8 and rgH9N2 virus as shown by 100% survival rates and relatively quicker recovery than the rgH3N2 group with 0 to 30% survival rates. Also, prime dose of rgH3N2 4xM2e provided higher protection with minimum weight loss against lethal challenge with H3N2 A/Phil, rgH5N1, and rgH7N9 virus although rgH3N2 primed mice survived in the absence of inducing cross reactive HAI activity. The efficacy of cross protection by priming with rgH3N2 4xM2e appears to be correlated with enhanced levels of M2e IgG antibodies in sera and mucosal (BALF, lungs) sites as well as IFN-γ secreting CD4 T cells (Fig. 5e,f).

The rgH3N2 control group showed substantial protection against H1N1, rgH5N1, rgH7N9, and rgH9N2 virus and A/Phil (H3N2) virus in the absence of cross-reactive HAI titers, lowering 100 folds in lung viral titers compared to mock control mice after infection. With CD4 or CD8 T cell depletion, the differences in cross protection against H3N2 A/Phil and H1N1 A/PR8 viruses were more evident between the rgH3N2 and rgH3N2 4xM2e primed mice. Also, the survival rates were lower particularly in the rgH3N2 group and recovery was delayed under a condition of both CD4 and CD8 T cell depletion even after low challenge doses with group 1 H1N1 virus A/PR8 and reassortants. These results support the significant roles of cross protective T cells particularly during a recovery phase. It is also possible that HA stalk specific antibodies induced in both chimeric 4xM2e-HA and WT rgH3N2 virus groups would contribute to cross protection against group 2 viruses with relatively high doses. This cross protection observed in the control rgH3N2 group, despite significantly lower efficacy than rgH3N2 4xM2e, indicates a caveat in testing vaccine efficacy of live recombinant influenza viruses in mouse models. Consistent, the mice surviving pathogenic influenza virus infection were reported to confer heterosubtypic cross protection^21,36,37^.

The levels of M2e specific IgG antibodies were significantly increased post boost with live rgH3N2 4xM2e virus, indicating effective priming of B cells by the first dose. In line with this outcome, enhanced cross protection against A/Phil H3N2 virus was observed in the rgH3N2 4xM2e group displaying no apparent weight loss after boost compared to the rgH3N2 control. Use of higher dose (50 LD_50_) challenge is because of prime boost vaccination and the same subtype as the rgH3N2 vaccine strain. When challenged with heterosubtypic rgH5N1 virus, the increases in M2e specific IgG responses in BALF and lungs but not IgG responses to rgH5N1 were higher than those with homosubtypic A/Phil H3N2 challenge. Consistently, BALF and lungs showed highly enhanced levels of IgG and IgA antibodies specific for M2e, compared to the control group.

A recent study reported that inactivated chimeric influenza viruses containing an M2e epitope in the immunodominant head site of HA could induce IgG antibodies to M2e and stalk domains after intramuscular vaccination, conferring cross protection^22,38^. The size of foreign epitopes or fragments to be inserted into the N-terminus of HA appears to be highly flexible as large as 246 residues while maintaining HA functional^39^. Whereas, recombinant influenza viruses containing only limited length of foreign epitopes less than 18 residues in the HA head domain could be rescued as for generating replication competent viruses^40,41^. The location of inserting foreign epitopes in HA molecules should be considered in the size of epitopes and routes of vaccination.

The non-neutralizing immune mechanisms of protection by M2e antibodies were reviewed^29,30,42^ in addition to M2e T cell contributions. Previous studies reported that M2e vaccine immune sera did not exhibit neutralization activity by plaque reduction or tissue culture infectivity assays^33,43^. Mechanisms of protection by passive transfer of M2e antibodies include antibody dependent cell-mediated cytotoxicity and antibody dependent cell-mediated phagocytosis, which involve Fc receptors, complements, natural killer (NK) cells, and macrophages. Passive transfer of M2e vaccine immune sera prior to virus infection provides survival advantages^44^. The infection of naïve mice with a mix of challenge virus and sera required smaller amounts of sera than the passive transfer of sera prior to infection. Our current and previous studies indicate that both simultaneous mix and prior-to-infection passive transfer approaches produced similar outcomes. We previously reported that the efficacy of M2e immune sera was significantly reduced or abrogated in Fcγ receptor knockout mice, suggesting a critical role of Fcγ receptors in mediating M2e antibody mediated protection^45–47^. M2e antibody-dependent NK cell activity was reported to be important for M2e immune mediated protection^33^.

In summary, recombinant seasonal influenza rgH3N2 4xM2e virus containing tandem repeat 4xM2e epitopes in the N-terminus of HA molecules retains comparable growth properties in vitro and LAIV-like attenuation phenotypes in vivo in mice. Intranasal prime vaccination with rgH3N2 4xM2e virus could provide broad and enhanced cross protection against different subtypes H1N1, H3N2, rgH5N1, rgH7N9, and rgH9N2 as well as reassortant viruses with avian M2. In vivo limited replication of recombinant influenza virus containing foreign epitopes in the N-terminus HA appears to be an attractive strategy to induce systemic and mucosal immune responses to the inserted epitopes. This study provides insight into developing broad cross protective recombinant influenza virus vaccines. This approach of recombinant influenza virus vaccine platforms with a licensed master backbone should be further tested in ferrets, a more relevant animal model in future studies.

## Materials and methods

### Cells and viruses

For DNA transfection to generate reassortants, 293 T cells obtained from ATCC were used and maintained in Dulbecco's Modified Eagle Medium media. Embryonated chicken eggs for influenza virus amplification were obtained from Hy-Line N.A. LLC (Mansfield, GA) and confirmed free of influenza virus. A/Puerto Rico/8/1934 (A/PR8, H1N1), A/Philippines/2/82 (A/Phil, H3N2), and rgH5N1 A/Vietnam virus which contains HA (polybasic residues removed) and NA from A/Vietnam/1203/2004 and 6 internal genes from A/PR8 were previously described^19^. Reverse genetics (rg) H3N2 (A/Switzerland/2013, A/SW) is a reassortant virus X-247 with A/PR8 backbone (CDC influenza resources) containing HA and NA genes from A/Switzerland /9715293/2013 (H3N2) and 6 remaining genes from A/PR8. The viruses were inactivated using formalin (37%) at 1:4000 dilutions in purified virus concentrations (1 mg/ml)^48^. The rgH7N9 is a reassortant virus containing HA and NA derived from A/Shanghai/2/2013 (H7N9) and the A/PR8 backbone genes^43^. The reassortant H9N2 virus is A/chicken/Hong Kong/G9/1997 × PR8-IBCDC-2 (CDC influenza resources). The rgPR8 Ms, rgPR8 Mm and rgPR8 Mg reassortant viruses containing avian M2 were generated using the A/PR8 backbone genes by replacing an M gene from A/Shanghai/2/2013 (H7N9), A/Mandarin duck/Korea/PSC24-24/2010 (H5N1) and A/Chicken/Korea/Gimje/2008 (H5N1) respectively.

### Generation of recombinant reassortant rgH3N2 virus containing 4xM2e-HA

A full-length copy of the HA gene from A/SW (H3N2) was used to insert a cloning site for BsmBI restriction enzyme between the signal peptide (SP) sequence and N-terminus in the ectodomain of HA by polymerase chain reaction (PCR) primers. The two PCR products of SP fragment and HA full length with each BsmBI site were linked together by overlapping reactions of PCR products, resulting in the SP-BsmBI-HA construct with an H3 HA full length ectodomain and transmembrane domain. The 4xM2e encoding gene is composed of heterologous M2e sequences derived from human (2xhM2e), swine (1xsM2e), and avian (1xaM2e) influenza A viruses (Fig. 1a) as described^21^. This 4xM2e gene was amplified using PCR primers with BsmBI sites for insertion into the SP-BsmBI-HA H3 construct in dual promoter pCI plasmid (Fig. 1a). The inserted 4xM2e gene contains flexible linkers AAAGGAA or AAAPGAA between each M2e domain as well as between the 4xM2e sequence and the N-terminus H3 HA (Fig. 1a). The correct insertion of 4xM2e into the full-length H3 HA was confirmed by DNA sequencing of the construct.

To generate reassortant rgH3N2 virus containing 4xM2e-HA (rgH3N2 4xM2e), 293 T cells were co-transfected with plasmids encoding 4xM2e-HA (H3) and N2 NA from A/SW (H3N2) and six other plasmids encoding the A/PR8 backbone genes. Two days after transfection, the supernatants were inoculated into 10-day-old embryonated chicken eggs. The rescue of replication competent recombinant viruses was initially screened in egg allantoic fluids by a hemagglutination activity assay and the genetic identity of the reassortant vaccine virus was confirmed by sequence analysis.

### Characterization and pathogenicity of reassortant rgH3N2 4xM2e-HA virus

The expression of 4xM2e-HA of the rescued reassortant viruses was characterized by enzyme-linked immunosorbent assay (ELISA) using influenza A virus M2e specific monoclonal antibody (14C2 mAb) (Abcam Inc., Cambridge, MA) or polyclonal antisera specific for H3N2 virus. Growth capability and kinetics of rescued rgH3N2 4xM2e virus were compared with rgH3N2 virus in embryonated eggs.

To verify pathogenicity of recombinant rgH3N2 4xM2e virus compared to A/PR8 pathogenic WT virus, BALB/c mice were intranasally (IN) inoculated with 1 × 10^5^ or 1 × 10^6^ EID_50_ (50% egg infectious dose) of rgH3N2 4xM2e, rgH3N2, or 10^4^ EID_50_ WT A/PR8 pathogenic viruses. At 3 days after IN infection, nasal turbinates and lung tissue samples were collected and homogenized in DMEM. The supernatants clarified by centrifugation were used to determine virus titers by limiting dilutions in the embryonated eggs and the limit of detection was 1.2 log_10_EID_50_/ml. Another set of mice (n = 5 /group) infected with recombinant and WT viruses was daily monitored for 14 days to record body weight changes.

### Immunizations and virus challenge of mice

BALB/c mice (n = 30 /group, 6- to 8-week-old age, Jackson Laboratories) were IN inoculated with 10^5^ EID_50_ for prime single dose of recombinant rgH3N2 4xM2e or rgH3N2 virus. Another set of primed mice (n = 15 /group) was boosted with 10^6^ EID_50_ of recombinant rgH3N2 4xM2e or rgH3N2 virus at 4 weeks after priming. Blood samples were collected at 2 weeks after each inoculation for serological immune assays. After 3 weeks of prime or prime boost dose inoculation, rgH3N2 4xM2e, rgH3N2 immune, or unvaccinated (mock) mice were challenged with different subtype viruses as indicated. LD_50_ dose causing 50% lethality in mice was pre-determined. Differential challenge doses were used depending on the pathogenicity and subtype of virus and experimental conditions (prime, prime-boost, T cell depletion). All animal experiments in this study were approved by the Georgia State University IACUC review boards. Mouse animal experiments including virus infection, blood and tissue collections were performed in accordance with the approved IACUC protocol (A21004) and regulations. The study was carried out in compliance with the ARRIVE (Animal Research: Reporting of In Vivo Experiments) guidelines.

### Antibody ELISA and hemagglutination inhibition (HAI) assays

Virus-specific antibody responses were determined by ELISA using inactivated H1N1 (A/PR8), H3N2 (A/SW, A/Phil), or rgH5N1 viruses as a coating antigen (4 µg/ml). M2e-specific antibody responses were determined using human, swine, and avian M2e peptide antigens of chemically synthesized 23 amino acids (aa) polypeptides as described^13,20^. IgG isotypes and IgA antibodies were measured using horse-radish peroxidase-conjugated goat anti-mouse IgG1, IgG2a, and IgA secondary antibodies (Southern Biotechnology), color reactions developed with tetramethylbenzidine substrates (TMB, Invitrogen). Antibody levels are presented as optical density absorbance values at 450 nm (BioTek ELISA plate reader) or concentrations as calculated using standard IgG and IgA (Southern Biotech). Bronchoalveolar lavage fluids (BALFs) were obtained by infusing 1.5 ml of phosphate-buffered saline (PBS) into lungs^13^. HAI assays were determined against homologous, heterologous, and heterosubtypic influenza viruses using chicken red blood cells^20,49^. Briefly, 1:4 ratios of sera and receptor destroying enzymes (RDE, Sigma) were mixed and incubated for 18 h at 37 °C. After inactivation, the RDE-treated sera were serially diluted and mixed with virus (8 HA units). HAI titers were determined as the highest dilution factor inhibiting the formation of buttons with 0.5% chicken red blood cells.

### In Vivo* T-cell depletion*

For in vivo systemic T-cell depletion prior to challenge, primed mice with rgH3N2 4xM2e or rgH3N2 received treatment with anti-CD4 (CD4 clone GK1.5) and/or anti-CD8 (CD8 clone 53.6.7) mAbs. Antibodies (BioXCell, West Lebanon, NH) were injected to the mice with intraperitoneal (IP) and IN sequential delivery at 2 days interval (anti-CD4 200 µg /mouse (IP) and 10 µg/mouse (IN), anti-CD8, 150 µg /mouse (IP), 10 µg/mouse (IN)). The levels of CD4 and CD8 T cells were below the detection after treating with CD4 and CD8 depleting antibodies as confirmed by flow cytometry of bloods (Supplementary Fig. S7). All groups (n = 4) were challenged with a lethal dose of A/PR8 H1N1 (1.5 LD_50_), rgPR8 Mg (3 LD_50_), rgPR8 Ms (10 LD_50_) or A/Phil H3N2 (17 LD_50_) influenza virus. Mice were monitored daily to record weight changes and mortality.

### Intracellular cytokine staining of T cells

BALF and lung cells were stimulated with M2e peptides (SLLTEVETPIRNEWGSRSN) (5 μg/mL) for 5 h at 37 °C in the presence of brefeldin A (BFA) (20 μg/mL). After stimulation, lymphocytes were stained with T cell marker mAb for CD4 (CD4-PE/Cy5, BD Biosciences) and CD8 (CD8α-PE, Biolegend) by following a procedure of BD Cytofix/Cytoperm Plus Kit. Intracellular staining of the permeabilized lymphocytes was conducted with IFN-γ cytokine mAb (anti-mouse IFN-γ-APC/Cy7, BD Biosciences). All samples were analyzed by using LSR-II/Fortessa flow cytometer (BD Biosciences, San Diego, CA, USA) and analyzed using the Flowjo software (FlowJo V10, Tree Star, Inc.).

### In vivo* efficacy tests of immune sera*

Immune sera collected at 4 weeks after boost inoculation were heat-inactivated at 56 °C for 30 min, 4 folds diluted, and then mixed with 2 LD_50_ of influenza virus rgH5N1 (A/Vietnam) and incubated at room temperature for 1 h as previously described^19,20^. The rgH5N1 virus mixed with immune sera was used to IN infect naive mice (n = 3) and body weight and survival rates were daily monitored for 8 days.

### In vitro* IgG antibody detection and cytokine ELISA assays*

Secreted IgG antibodies specific for M2e, rgH3N2 and A/Phil were determined from mediastinal lymph nodes (MLN) from BALB/c mice. The cells from MLN were isolated at day 6 after challenge and cultured for 6 days in the plate pre-coated with M2e, rgH3N2 or A/Phil. The combined levels of IgG antibodies secreted into the culture supernatants and captured on the plate were analyzed by ELISA and presented. The inflammatory cytokines such as TNF-α, IFN-γ and IL-6 from bronchoalveolar lavage fluids (BALF) and lung extracts were measured by cytokine ELISA as described in the previous study^50^. Cytokines were detected using Ready-SET-Go kits with TNF-α, IFN-γ or IL-6 specific antibodies (eBioscience, San Diego, CA).

### Statistical analysis

Two-way or one-way ANOVA were used to determine the statistical significance when comparing two different conditions. *P*-values of less than or equal to 0.05 were considered significant. Data analysis was performed using Prism software (GraphPad software Inc., San Diego, CA).

## Supplementary Information

Supplementary Information.
