# Supplementary material for: Broad cross protection by recombinant live attenuated influenza H3N2 seasonal virus expressing conserved M2 extracellular domain in a chimeric hemagglutinin
Source: Sci Rep. 2021 Feb 18;11:4151. doi: 10.1038/s41598-021-83704-0 (PMC7893060; doi:10.1038/s41598-021-83704-0)
Supplement: Supplementary file 1 — Supplementary Information. [file 41598_2021_83704_MOESM1_ESM.pdf]

Broad cross protection by recombinant live attenuated influenza H3N2 seasonal virus expressing conserved M2 extracellular domain in a chimeric hemagglutinin

Bo Ryoung Park[1], Ki-Hye Kim[1], Tatiana Kotomina[2], Min-Chul Kim[1,3], Young-Man Kwon[1], Subbiah Jeeva[1], Yu-Jin Jung[1], Noopur Bhatnagar[1], Irina Isakova-Sivak[2], Daria Mezhenskaya[2], Larisa Rudenko[2], Bao-Zhong Wang[1] Sang-Moo Kang[1, *]

Vaccine virus (rgH3N2 4xM2e)

Conserved M2e	M2e sequences
Human M2e	SLLTEVET**PIRNEW**GSRSNDSSD
Swine M2e	SLLTEVET**PTRSEW**ESRSSDSSD
Avian M2e	SLLTEVET**PTRNEW**ESRSSDSSD

Challenge virus

M2 gene from	M2e sequences
A/Puerto Rico/8/1934 (H1N1)	SLLTEVET**PIRNEW**GCRCN GSSD
A/California/07/2009 (H1N1)	SLLTEVET**PTRSEW**ECRCSDSSD
A/Philippines/2/82 (H3N2)	SLLTEVET**PIRNEW**GCRCN DSSD
A/Madarin duck/Korea/PSC24-24/2010 (H5N1)	SLLTEVET**PTRNEW**ECRCSDSSD
A/Chicken/Korea/Gimje/2008 (H5N1)	SLLTEVET**PTRHEW**ECRCSDSSD
A/Shanghai/2/2013 (H7N9)	SLLTEVET**PTRTGW**ECNCSGSSE

Supplementary Table S1. M2e sequences utilized for rgH3N2 4xM2e vaccine and reassortant challenge viruses.

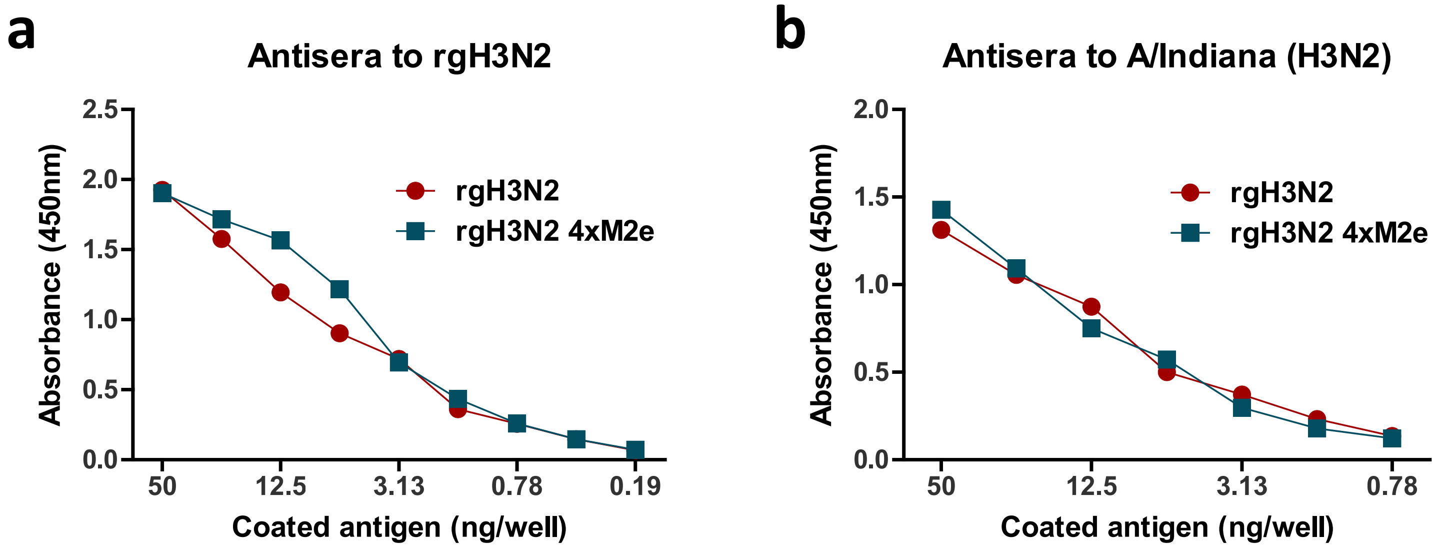

Supplementary Figure S1. ELISA assays to characterize 6:2 reassortants rgH3N2 and rgH3N2 4xM2e viruses generated by reverse genetics (rg) using the A/PR8 backbone.

(a) ELISA (4 µg/ ELISA plate well) using anti-rgH3N2 (A/Switzerland/9715293/2013 (H3N2)) mouse sera as primary antibody. (b) ELISA using anti-A/Indiana/2011 (H3N2) goat sera as primary antibody.

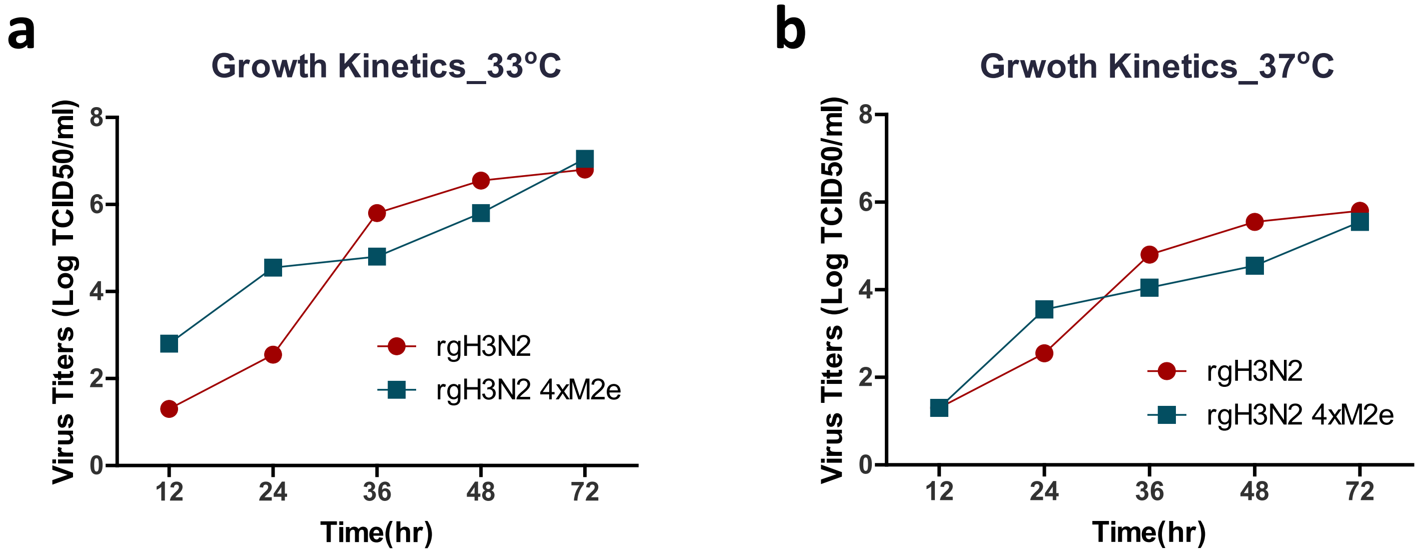

Supplementary Figure S2. Growth kinetics for rgH3N2 and rgH3N2 4xM2e in MDCK cells.

Virus replications of rgH3N2 and rgH3N2 4xM2e were determined in MDCK cells at different time points at 33ºC and 37ºC culture temperature as indicated after inoculation. The titers were determined and presented in $TCID_{50}$/ml (a) at 33°C and (b) at 37°C.

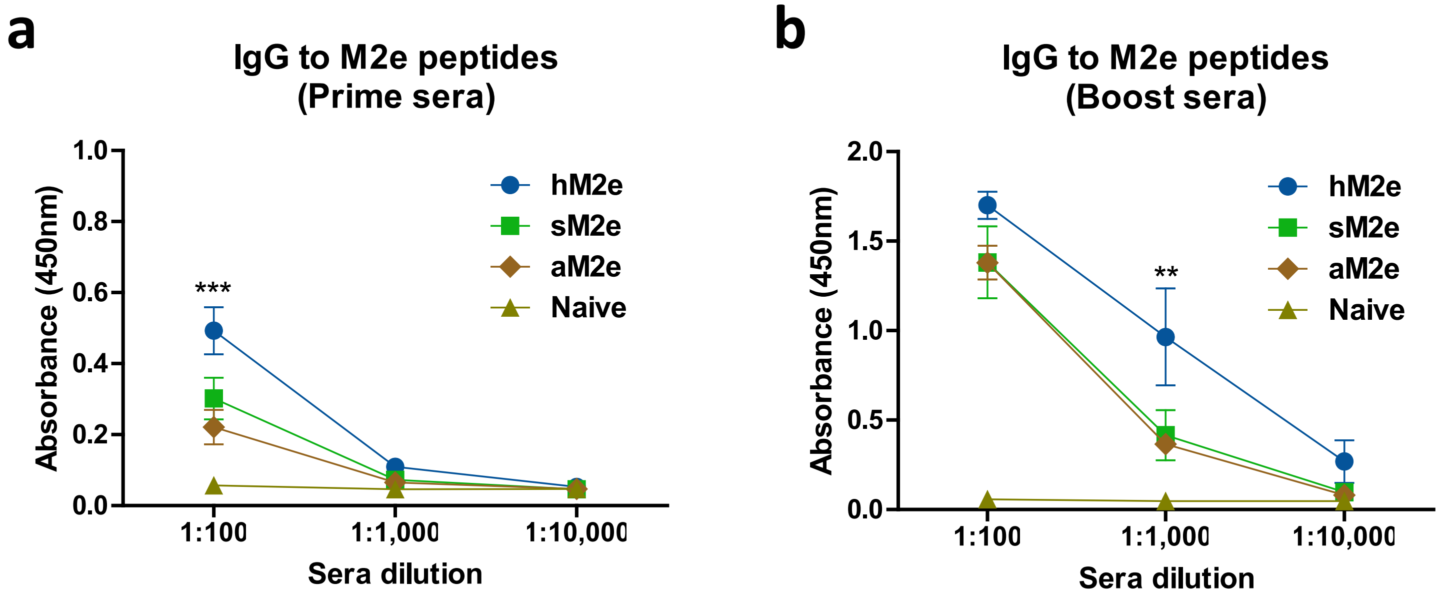

Supplementary Figure S3. IgG antibody responses to humanM2e, swine M2e, and avian M2e peptides after prime or prime boost vaccination with rgH3N2 4xM2e.
Mouse sera were collected 2 weeks after intranasal prime or prime boost inoculation of rgH3N2 4xM2e. Total IgG antibody specific for human M2e (hM2e), avian M2e (aM2e) and swine M2e (sM2e) were measured from (a) prime and (b) boost sera. Error bars show mean ± SEM. The statistical significances between hM2e detection versus sM2e and aM2e were determined using two-way ANOVA and indicated in **, $P < 0.01$; ***, $P < 0.001$.

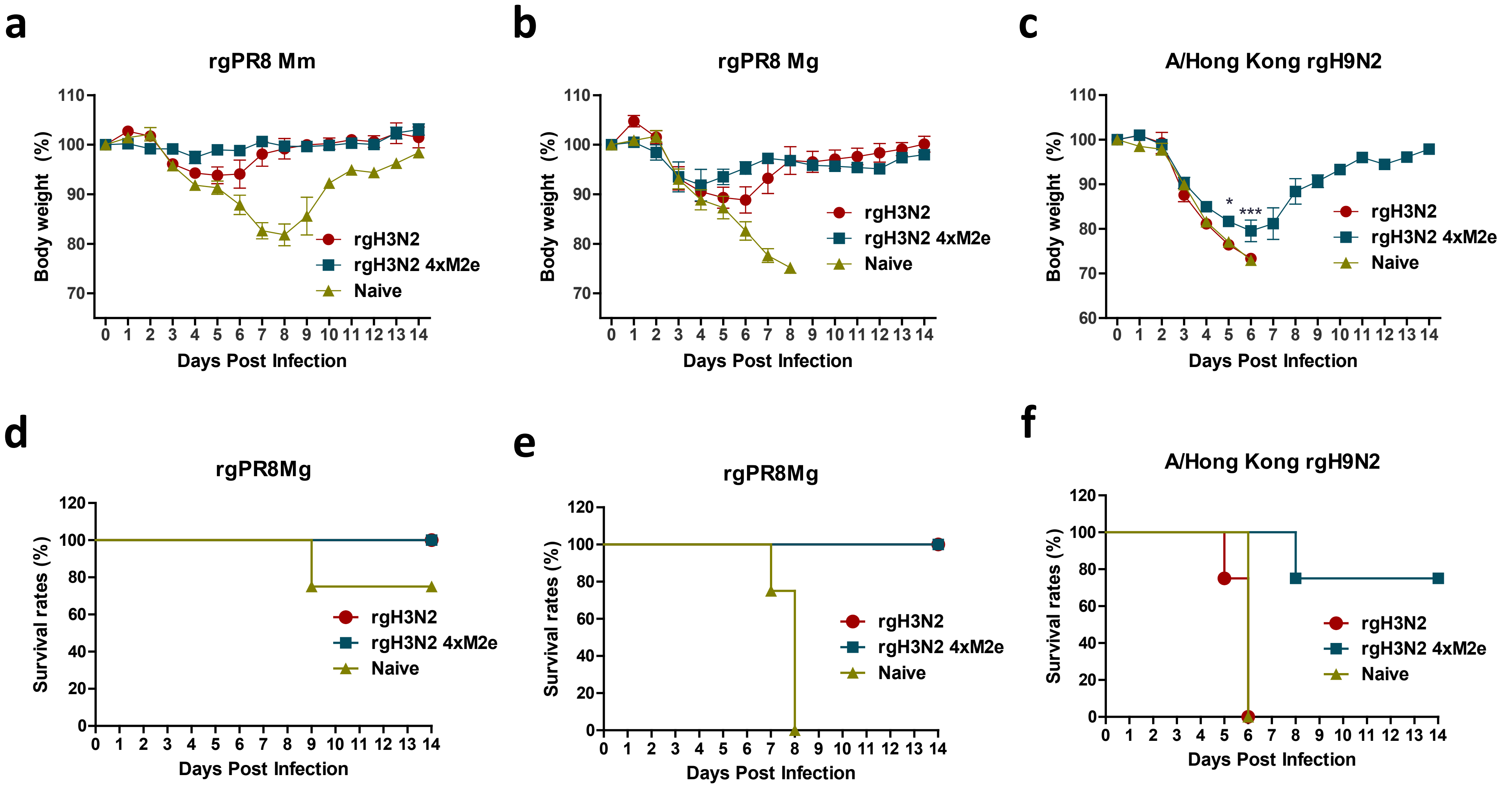

Supplementary Figure S4. A single dose of live rgH3N2 4xM2e virus provides enhanced heterosubtypic cross protection against A/PR8 (H1N1) reassortants containing avian M gene or rgH9N2 virus.

(a-c) Weight changes and (d-f) survival rates after challenge with rgPR8 Mm (M gene from A/Mandarin duck/Korea/PSC24-242010, H5N1, 1 LD_{50}), rgPR8 Mg (M gene from A/Chicken/Korea/Gimje/2008, H5N1, 3 LD_{50}) and high dose of rgH9N2 virus (reassortant A/Hong Kong, 100 LD_{50}), respectively. Groups of mice (n=3 or 4) were intranasally primed with rgH3N2 (10^5 EID_{50}) or rgH3N2 4xM2e (10^5 EID_{50}) and then challenged 3 weeks later. Error bars show mean ± SEM. The statistical significances between rgH3N2 group versus rgH3N2 4xM2e group were determined using two-way ANOVA and indicated in *, $P < 0.05$; **, $P < 0.01$; ***, $P < 0.001$.

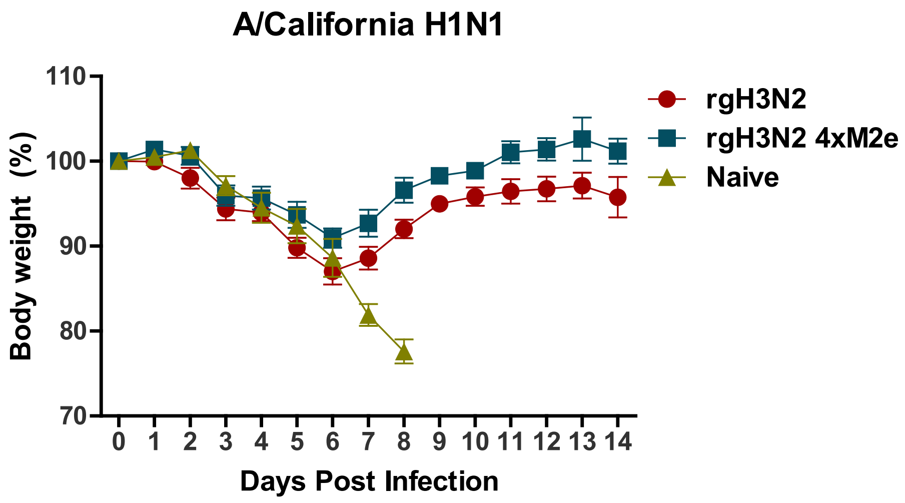

Supplementary Figure S5. Body weight changes in BALB/c mice after challenge with A/California virus containing swine M2.

The groups of BALB/c mice were infected with A/California/04/09 (6 LD_{50}) containing swine M2 at 3 weeks after single dose intranasal immunization with rgH3N2 or rgH3N2 4xM2e (10^5 EID_{50} each).

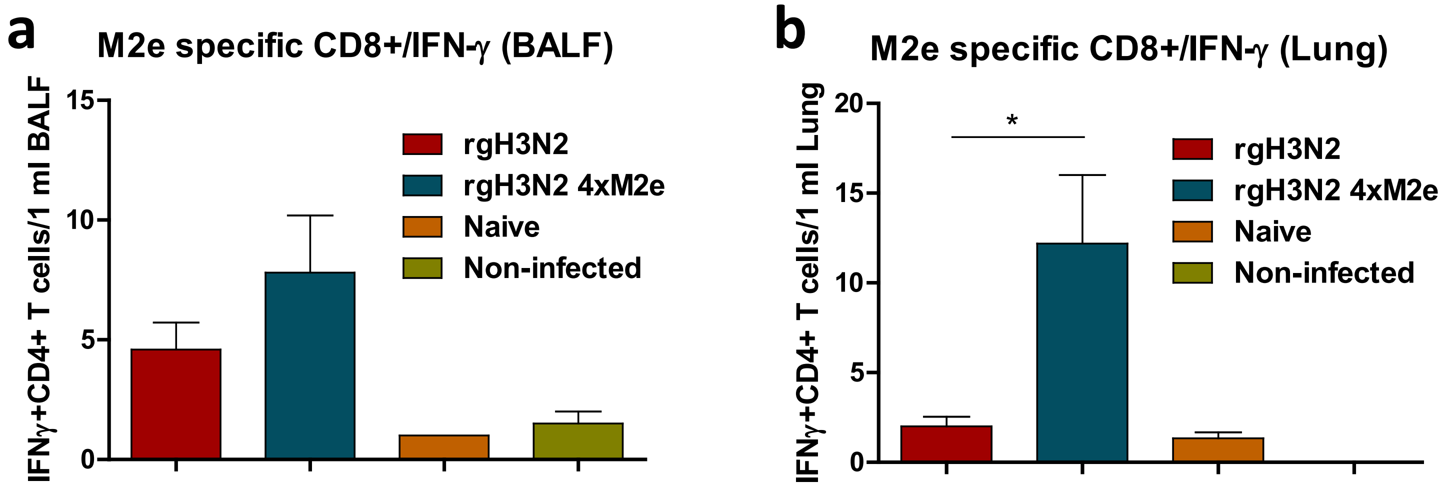

Supplementary Figure S6. Measurement of M2e specific CD8^{+} cells secreting IFN-γ by flow cytometry.
BALB/c mice were intranasally inoculated with rgH3N2 or rgH3N2 4xM2e (10^5 EID_{50} each). IFN-γ secreting CD8^{+} cells specific for M2e were detected 5 days after A/Phil challenge (50 LD_{50}) in BALF (a) and lung cells (b) from immunized mice. IFN-γ^{+} CD4^{+} T cells were presented from total BALF (1 ml) and Lung (1 ml) cells from individual mouse. Error bars indicate mean ± SEM. The statistical significances between rgH3N2 group versus rgH3N2 4xM2e group were determined using one-way ANOVA and indicated in *, $P < 0.05$.

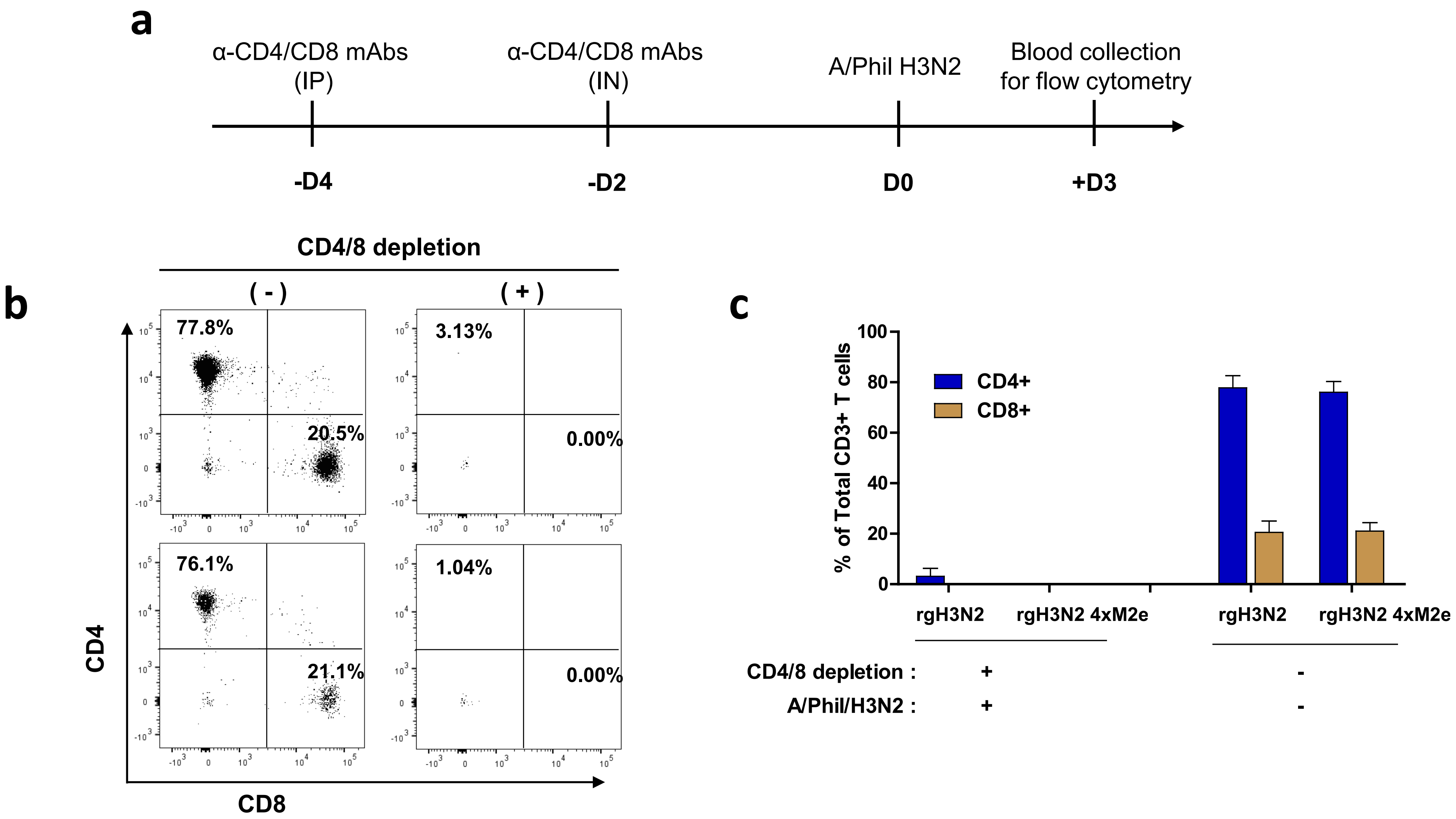

Supplementary Figure S7. The efficacy of CD4 and CD8 T cell depletion in vaccinated mice after CD4 and CD8 T cell depleting antibody treatment.
(a) Schematic diagram for CD4 and CD8 T cell depleting antibody treatment schedule. (b) Flow cytometry profiles of $CD4^+$ and $CD8^+$ T cells out of total $CD3^+$ T cells in peripheral blood mononucleocytes (PBMC). (c) Percentages of $CD4^+$ T cells out of $CD3^+$ cells, and $CD8^+$ T cells out of total $CD3^+$ cells in PBMC. rgH3N2: the group of mice vaccinated with rgH3N2 (n=3). rgH3N2 4xM2e: the group of mice vaccinated with rgH3N2 4xM2e (n=3).

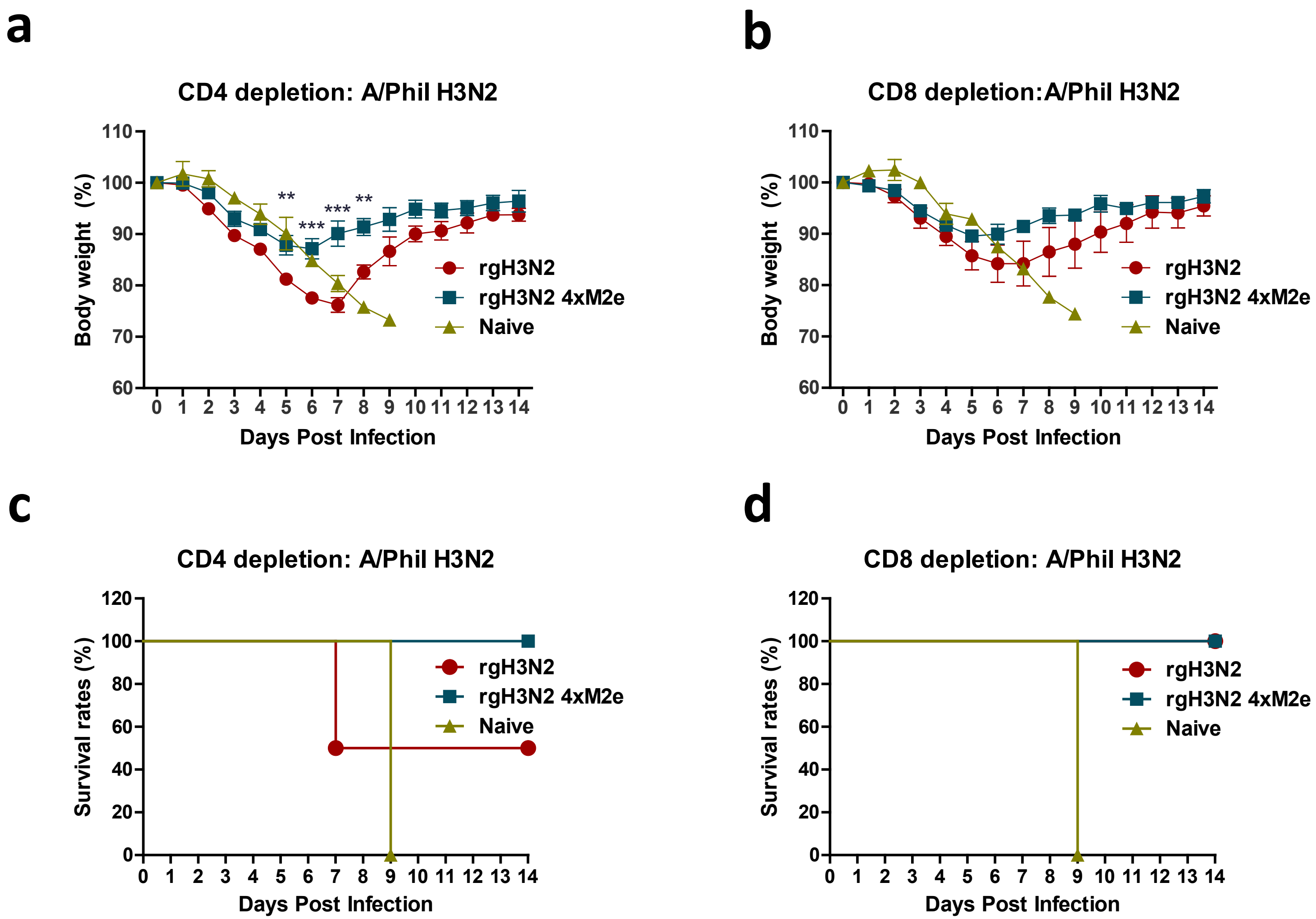

Supplementary Figure S8. The roles of CD4$^+$ and CD8$^+$ T cells in cross protection by reassortant rgH3N2 4xM2e prime inoculation.
The rgH3N2 or rgH3N2 4xM2e primed mice (n=4 /group) with were treated with (a, c) α-CD4 or (b, d) α-CD8 antibodies for T cell depletion prior to A/Phil (H3N2) challenge (25 LD_{50}). Weight changes and survival rates were monitored after A/Phil challenge. Error bars indicate mean value ± SEM. The statistical significances between rgH3N2 group and rgH3N2 4xM2e group were determined using two-way ANOVA and indicated in *, $P < 0.05$; **, $P < 0.01$; ***, $P < 0.001$.

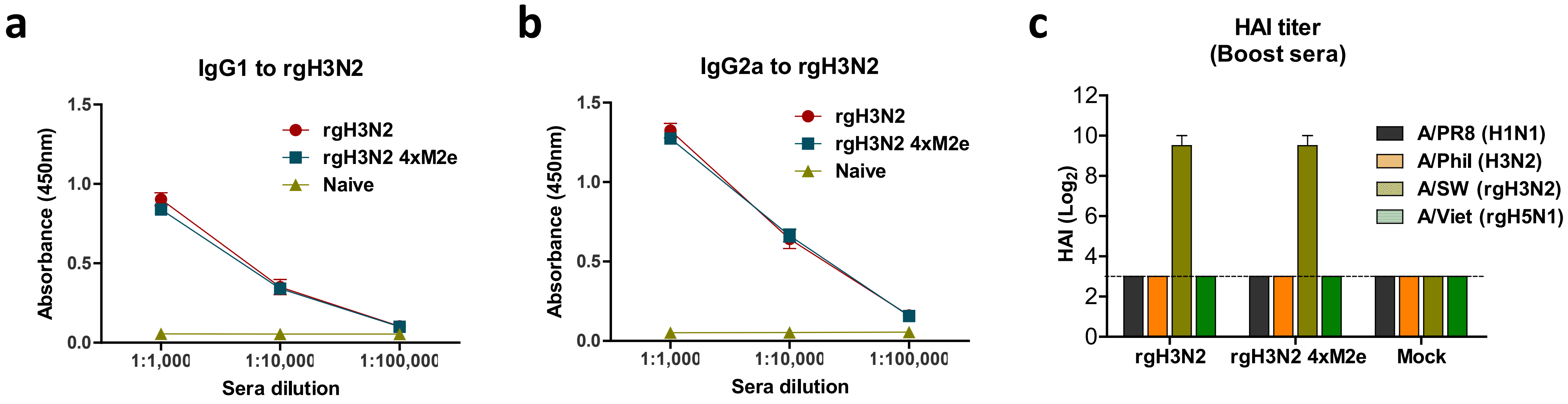

Supplementary Figure S9. Intranasal boost vaccination with live rgH3N2 4xM2e virus further enhances rgH3N2 virus specific IgG and HAI antibodies. Sera were collected at 2 weeks after boost immunization of primed mice with rgH3N2 or rgH3N2 4xM2e. (a) IgG1 or (b) IgG2a antibody detection specific for rgH3N2. (c) Boost serum HAI titers against A/PR8, A/Phil, rgH3N2 and A/Viet. Error bars indicate mean ± SEM.
